# Supplementary material for: Agonists of Orally Expressed TRP Channels Stimulate Salivary Secretion and Modify the Salivary Proteome
Source: Mol Cell Proteomics. 2020 Nov 25;19(10):1664–76. doi: 10.1074/mcp.RA120.002174 (PMC8014997; doi:10.1074/mcp.RA120.002174)
Supplement: Supplementary file 1 [file mmc1.zip › 161586_0_supp_549148_qc0vbt.pdf]

**Supplementary Information**

Novel data analysis method

The following process was applied to each batch separately: peptides were excluded if they were unassigned or had missing TMT channel intensity data; the primary accession number was taken for each peptide and proteins were grouped by this accession number with the geomean of individual peptide intensities given as the protein intensity value; TMT intensities were normalised using a sum scaling method. PCA was carried out on each batch to obtain a 3-dimensional coordinate for each sample within the batch, with the value of each dimension value being normalised by the eigenvalue for that dimension. Euclidean distances were calculated, using an R package, between each sample and the relevant standard pool sample for that participant. Euclidean distances between samples and unstimulated pools were normalised to the Euclidean distance between the two standard pools in the batch. Batches were then concatenated for analysis by PCA.

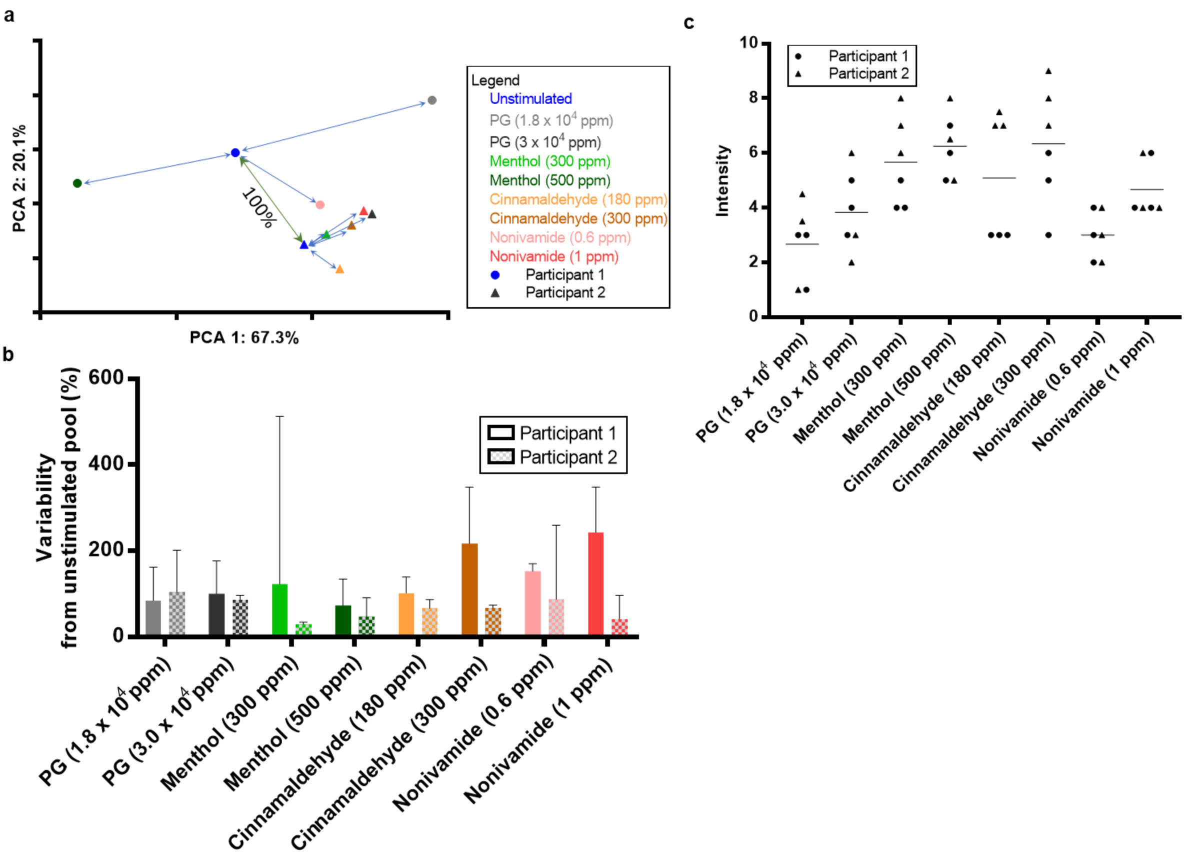


Supplementary Figure: Identification of inter-day variation using a novel bioinformatic method

a) PCA plot showing the Euclidean distances between single post-mouth rinse coordinates and their relevant unstimulated pool standard (blue lines) coordinate and the normalising Euclidean distance between the two unstimulated pool standard (green line) coordinates within one batch.

b) Histogram of Euclidean distances between post-mouth rinse coordinates and the unstimulated pool standard relative to the Euclidean distance between the two unstimulated pool standards which is set at 100% (mean ±SEM); $n$ = 3).

c) Intensity ratings reported on a visual analogue scale for the administered TRP channel agonist and control mouth rinses. Horizontal lines indicate the mean rating across both participants.

Supplementary Table

UniProt reviewed proteins identified in this study that have not previously been reported in two databases of human WMS.

| UniProt ID | Protein name | # batch's present | Relative abundance (%) | Mean coverage (%) |
|---|---|---|---|---|
| Q9UGM3 | Deleted in malignant brain tumors 1 protein | 6 | 3.2 | 30.5 |
| P01591 | Immunoglobulin J chain | 6 | 1.2 | 53.9 |
| A0A0C4DH25 | Immunoglobulin kappa variable 3D-20 | 4 | 0.0 | 7.8 |
| P01619 | Immunoglobulin kappa variable 3-20 | 3 | 0.2 | 16.7 |
| P01714 | Immunoglobulin lambda variable 3-19 | 2 | 0.0 | 16.7 |
| O60673 | DNA polymerase zeta catalytic subunit | 1 | 0.0 | 3.1 |
| P12107 | Collagen alpha-1 | 1 | 0.0 | 0.5 |
| Q92766 | Ras-responsive element-binding protein 1 | 6 | 0.2 | 0.8 |
| Q8NBJ4 | Golgi membrane protein 1 | 3 | 0.0 | 3.0 |
| A0A0C4DH31 | Immunoglobulin heavy variable 1-18 | 2 | 0.0 | 10.7 |
| Q96MD7 | Uncharacterized protein C9orf85 | 4 | 0.1 | 4.5 |
| Q9NSI6 | Bromodomain and WD repeat-containing protein 1 | 3 | 0.1 | 1.5 |
| P04433 | Immunoglobulin kappa variable 3-11 | 6 | 0.1 | 13.0 |
| P06396 | Gelsolin | 6 | 0.2 | 2.5 |
| Q9BWT7 | Caspase recruitment domain-containing protein 10 | 6 | 0.3 | 1.5 |
| P01717 | Immunoglobulin lambda variable 3-25 | 5 | 0.1 | 17.8 |
| P01766 | Immunoglobulin heavy variable 3-13 | 5 | 0.3 | 15.8 |
| P80748 | Immunoglobulin lambda variable 3-21 | 5 | 0.1 | 7.2 |
| Q5JU00 | Dynein regulatory complex subunit 5 | 5 | 0.2 | 3.6 |
| P15311 | Ezrin | 5 | 0.0 | 2.2 |
| Q9NPA5 | Zinc finger protein 64 homolog, isoforms 1 and 2 | 5 | 0.0 | 1.7 |
| O95789 | Zinc finger MYM-type protein 6 | 5 | 0.3 | 1.0 |
| Q9BXW9 | Fanconi anemia group D2 protein | 5 | 0.1 | 0.7 |
| P13929 | Beta-enolase | 4 | 0.0 | 7.2 |
| Q8NHS2 | Putative aspartate aminotransferase, cytoplasmic 2 | 4 | 0.0 | 2.3 |
| Q147X3 | N-alpha-acetyltransferase 30 | 4 | 0.2 | 2.2 |
| Q8WUB2 | Protein FAM216A | 3 | 0.0 | 7.1 |
| A8MU10 | Putative uncharacterized protein ENSP00000381562 | 3 | 0.1 | 6.2 |
| P21912 | Succinate dehydrogenase [ubiquinone] iron-sulfur subunit, mitochondrial | 3 | 0.0 | 3.9 |
| Q6ZU35 | Uncharacterized protein KIAA1211 | 3 | 0.0 | 3.5 |
| Q9UN86 | Ras GTPase-activating protein-binding protein 2 | 3 | 0.2 | 2.8 |
| P15260 | Interferon gamma receptor 1 | 3 | 0.0 | 2.4 |
| O43184 | Disintegrin and metalloproteinase domain-containing protein 12 | 3 | 0.2 | 1.7 |
| Q9P2R6 | Arginine-glutamic acid dipeptide repeats protein | 3 | 0.0 | 1.5 |
| O60346 | PH domain leucine-rich repeat-containing protein phosphatase 1 | 3 | 0.2 | 0.8 |
| Q9BYB0 | SH3 and multiple ankyrin repeat domains protein 3 | 3 | 0.1 | 0.8 |
| Q03001 | Dystonin | 3 | 0.0 | 0.5 |

| | | | | |
|---|---|---|---|---|
| P01614 | Immunoglobulin kappa variable 2D-40 | 2 | 0.0 | 17.4 |
| P01762 | Immunoglobulin heavy variable 3-11 | 2 | 0.0 | 15.6 |
| P04406 | Glyceraldehyde-3-phosphate dehydrogenase | 2 | 0.0 | 11.3 |
| P62805 | Histone H4 | 2 | 0.0 | 6.8 |
| Q5SY16 | Polynucleotide 5'-hydroxyl-kinase NOL9 | 2 | 0.1 | 3.4 |
| Q8NA70 | Protein FAM47B | 2 | 0.0 | 2.9 |
| O43639 | Cytoplasmic protein NCK2 | 2 | 0.0 | 2.0 |
| P02679 | Fibrinogen gamma chain | 2 | 0.0 | 2.0 |
| Q7Z5J8 | Ankyrin and armadillo repeat-containing protein | 2 | 0.0 | 1.7 |
| Q9H0R5 | Guanylate-binding protein 3 | 2 | 0.0 | 1.6 |
| Q9Y2E4 | Disco-interacting protein 2 homolog C | 2 | 0.0 | 1.1 |
| Q4G0P3 | Hydrocephalus-inducing protein homolog | 2 | 0.0 | 0.9 |
| Q96BY7 | Autophagy-related protein 2 homolog B | 2 | 0.0 | 0.9 |
| Q5VT25 | Serine/threonine-protein kinase MRCK alpha | 2 | 0.0 | 0.5 |
| P29597 | Non-receptor tyrosine-protein kinase TYK2 | 2 | 0.0 | 0.4 |
| Q9BQS7 | Hephaestin | 2 | 0.1 | 0.4 |
| Q96EU6 | Ribosomal RNA processing protein 36 homolog | 1 | 0.0 | 12.0 |
| P09630 | Homeobox protein Hox-C6 | 1 | 0.0 | 10.2 |
| Q96M15 | Putative uncharacterized protein IGF2BP2-AS1 | 1 | 0.0 | 9.1 |
| Q9P291 | Armadillo repeat-containing X-linked protein 1 | 1 | 0.0 | 8.4 |
| Q9C0B5 | Palmitoyltransferase ZDHHC5 | 1 | 0.0 | 7.4 |
| Q9Y3M8 | StAR-related lipid transfer protein 13 | 1 | 0.0 | 6.7 |
| Q96HE9 | Proline-rich protein 11 | 1 | 0.0 | 6.4 |
| P02675 | Fibrinogen beta chain [Cleaved into: Fibrinopeptide B; Fibrinogen beta chain] | 1 | 0.0 | 6.1 |
| Q7L273 | BTB/POZ domain-containing protein KCTD9 | 1 | 0.0 | 5.9 |
| A6NIM6 | Solute carrier family 15 member 5 | 1 | 0.0 | 5.5 |
| P35052 | Glypican-1 [Cleaved into: Secreted glypican-1] | 1 | 0.0 | 5.2 |
| Q8N264 | Rho GTPase-activating protein 24 | 1 | 0.0 | 4.7 |
| P11142 | Heat shock cognate 71 kDa protein | 1 | 0.0 | 4.5 |
| Q9UPQ9 | Trinucleotide repeat-containing gene 6B protein | 1 | 0.0 | 4.5 |
| P55210 | Caspase-7 | 1 | 0.0 | 3.6 |
| Q13424 | Alpha-1-syntrophin | 1 | 0.0 | 3.6 |
| Q9BXM7 | Serine/threonine-protein kinase PINK1, mitochondrial | 1 | 0.0 | 3.6 |
| Q96IW2 | SH2 domain-containing adapter protein D | 1 | 0.0 | 3.2 |
| Q9UNA1 | Rho GTPase-activating protein 26 | 1 | 0.0 | 3.0 |
| Q8TF62 | Probable phospholipid-transporting ATPase IM | 1 | 0.0 | 2.9 |
| Q14185 | Dedicator of cytokinesis protein 1 | 1 | 0.0 | 2.8 |
| Q9BQI6 | SMC5-SMC6 complex localization factor protein 1 | 1 | 0.0 | 2.8 |
| P54652 | Heat shock-related 70 kDa protein 2 | 1 | 0.0 | 2.7 |
| Q6NS38 | DNA oxidative demethylase ALKBH2 | 1 | 0.1 | 2.7 |
| Q8IYB7 | DIS3-like exonuclease 2 | 1 | 0.0 | 2.6 |
| Q8TDG4 | Helicase POLQ-like | 1 | 0.0 | 2.6 |
| P38398 | Breast cancer type 1 susceptibility protein | 1 | 0.1 | 2.5 |
| P28799 | Granulins | 1 | 0.0 | 2.4 |
| Q9H0C8 | Integrin-linked kinase-associated serine/threonine phosphatase 2C | 1 | 0.0 | 2.3 |

| | | | | |
|---|---|---|---|---|
| Q86XI8 | Uncharacterized protein ZSWIM9 | 1 | 0.0 | 2.2 |
| Q15406 | Nuclear receptor subfamily 6 group A member 1 | 1 | 0.0 | 2.1 |
| Q9UKP6 | Urotensin-2 receptor | 1 | 0.0 | 2.1 |
| Q9Y4D1 | Disheveled-associated activator of morphogenesis 1 | 1 | 0.0 | 2.1 |
| Q0VFZ6 | Coiled-coil domain-containing protein 173 | 1 | 0.0 | 2.0 |
| Q8NG48 | Protein Lines homolog 1 | 1 | 0.0 | 1.9 |
| Q9UK32 | Ribosomal protein S6 kinase alpha-6 | 1 | 0.0 | 1.9 |
| O43847 | Nardilysin | 1 | 0.0 | 1.7 |
| P28370 | Probable global transcription activator SNF2L1 | 1 | 0.0 | 1.7 |
| Q8WYB5 | Histone acetyltransferase KAT6B | 1 | 0.0 | 1.7 |
| Q92800 | Histone-lysine N-methyltransferase EZH1 | 1 | 0.0 | 1.7 |
| Q9Y2W1 | Thyroid hormone receptor-associated protein 3 | 1 | 0.0 | 1.7 |
| O75367 | Core histone macro-H2A.1 | 1 | 0.0 | 1.6 |
| P13942 | Collagen alpha-2 | 1 | 0.0 | 1.6 |
| Q68DX3 | FERM and PDZ domain-containing protein 2 | 1 | 0.0 | 1.5 |
| Q68D51 | DENN domain-containing protein 2C | 1 | 0.1 | 1.4 |
| O60241 | Adhesion G protein-coupled receptor B2 | 1 | 0.0 | 1.3 |
| Q8TET4 | Neutral alpha-glucosidase C | 1 | 0.0 | 1.3 |
| Q6PJP8 | DNA cross-link repair 1A protein | 1 | 0.0 | 1.2 |
| Q9NVE7 | Pantothenate kinase 4 | 1 | 0.0 | 1.2 |
| Q9UG01 | Intraflagellar transport protein 172 homolog | 1 | 0.0 | 1.2 |
| A8MXQ7 | Putative IQ motif and ankyrin repeat domain-containing protein LOC642574 | 1 | 0.0 | 1.0 |
| O14936 | Peripheral plasma membrane protein CASK | 1 | 0.0 | 1.0 |
| P51532 | Transcription activator BRG1 | 1 | 0.0 | 1.0 |
| Q5JSZ5 | Protein PRRC2B | 1 | 0.0 | 1.0 |
| Q5T1R4 | Transcription factor HIVEP3 | 1 | 0.0 | 1.0 |
| Q659C4 | La-related protein 1B | 1 | 0.0 | 1.0 |
| Q86W92 | Liprin-beta-1 | 1 | 0.1 | 1.0 |
| O00566 | U3 small nucleolar ribonucleoprotein protein MPP10 | 1 | 0.0 | 0.9 |
| Q5CZC0 | Fibrous sheath-interacting protein 2 | 1 | 0.0 | 0.9 |
| Q5T5P2 | Sickle tail protein homolog | 1 | 0.0 | 0.9 |
| Q96C24 | Synaptotagmin-like protein 4 | 1 | 0.1 | 0.9 |
| Q9H4D0 | Calsyntenin-2 | 1 | 0.1 | 0.9 |
| O94986 | Centrosomal protein of 152 kDa | 1 | 0.0 | 0.8 |
| Q5JPF3 | Ankyrin repeat domain-containing protein 36C | 1 | 0.0 | 0.8 |
| P35916 | Vascular endothelial growth factor receptor 3 | 1 | 0.0 | 0.7 |
| P51825 | AF4/FMR2 family member 1 | 1 | 0.0 | 0.7 |
| Q7RTX0 | Taste receptor type 1 member 3 | 1 | 0.0 | 0.7 |
| Q86XK2 | F-box only protein 11 | 1 | 0.0 | 0.7 |
| O15050 | TPR and ankyrin repeat-containing protein 1 | 1 | 0.0 | 0.6 |
| P46100 | Transcriptional regulator ATRX | 1 | 0.0 | 0.6 |
| Q03164 | Histone-lysine N-methyltransferase 2A | 1 | 0.1 | 0.6 |
| Q86UW6 | NEDD4-binding protein 2 | 1 | 0.0 | 0.6 |
| Q96RL7 | Vacuolar protein sorting-associated protein 13A | 1 | 0.0 | 0.6 |
| A6NM11 | Leucine-rich repeat-containing protein 37A2 | 1 | 0.0 | 0.5 |
| Q96M86 | Dynein heavy chain domain-containing protein 1 | 1 | 0.1 | 0.5 |

| | | | | |
|---|---|---|---|---|
| Q9ULD4 | Bromodomain and PHD finger-containing protein 3 | 1 | 0.0 | 0.5 |
| Q5VYK3 | Proteasome adapter and scaffold protein ECM29 | 1 | 0.0 | 0.4 |
| Q96JQ0 | Protocadherin-16 | 1 | 0.0 | 0.4 |
| Q02224 | Centromere-associated protein E | 1 | 0.0 | 0.3 |
| Q12873 | Chromodomain-helicase-DNA-binding protein 3 | 1 | 0.0 | 0.3 |
| Q86WI1 | Fibrocystin-L | 1 | 0.1 | 0.3 |
